# The Relationships between Human Fatigue and Public Health: A Brief Commentary on Selected Papers from the 9th International Conference on Managing Fatigue in Transportation, Resources and Health

**DOI:** 10.3390/ijerph13090842

**Published:** 2016-08-24

**Authors:** Charli Sargent, Paul Roberts, Drew Dawson, Sally Ferguson, Lynn Meuleners, Libby Brook, Gregory D. Roach

**Affiliations:** 1 Appleton Institute for Behavioural Science, Central Queensland University, P.O. Box 42, Goodwood, SA 5034, Australia; drew.dawson@cqu.edu.au (D.D.); sally.ferguson@cqu.edu.au (S.F.); greg.roach@cqu.edu.au (G.D.R.); 2 ARRB Group Ltd., 191 Carr Place, Leederville, WA 6007, Australia; paul.roberts@arrb.com.au; 3 Curtin Monash Accident Research Centre, Curtin University, GPO Box U1987, Perth, WA 6845, Australia; l.meuleners@curtin.edu.au; 4 School of Psychology and Exercise Science, Murdoch University, 90 South Street, Murdoch, WA 6150, Australia; l.brook@murdoch.edu.au

**Keywords:** shiftwork, cardiovascular disease, sleep disordered breathing, eating behaviour, fatigue risk management systems

## Abstract

The 9th International Conference on Managing Fatigue in Transportation, Resources and Health was held in Fremantle, Western Australia in March 2015. The purpose of the conferences in this series is to provide a forum for industry representatives, regulators, and scientists to discuss recent advances in the field of fatigue research. We have produced a Special Issue of the *International Journal of Environmental Research and Public Health* based on papers from the conference that were focused on various aspects of public health. First, the Special Issue highlights the fact that working long shifts and/or night shifts can affect not only cognitive functioning, but also physical health. In particular, three papers examined the potential relationships between shiftwork and different aspects of health, including the cardiovascular system, sleep disordered breathing, and eating behaviour. Second, the Special Issue highlights the move away from controlling fatigue through prescriptive hours of service rules and toward the application of risk management principles. In particular, three papers indicated that best-practice fatigue risk management systems should contain multiple redundant layers of defense against fatigue-related errors and accidents.

## Editorial

The 9th International Conference on Managing Fatigue in Transportation, Resources and Health was held in Fremantle, Western Australia in March 2015. The purpose of the conference, which was first convened by Laurence Hartley in 1993, is to provide a forum in which industry representatives, regulators, and scientists can discuss recent advances in the field of fatigue research. This Special Issue of the *International Journal of Environmental Research and Public Health* contains papers from the conference that were focused on various aspects of public health.

Elliot and Lal [1] examined the impact of shiftwork on cardiovascular health with 206 general duties police officers. Each participant’s blood pressure was assessed before and after either a 12-h day shift (06:00–18:00, *n* = 92) or a 12-h night shift (18:00–06:00, *n* = 114). For female participants (*n* = 66), blood pressure was higher at the end of a shift than at the start. There was no difference in pre- and post-shift blood pressure for male participants (*n* = 140). The analyses did not include shift type (day vs. night) or time of day (06:00 vs. 18:00), so the potentially important interactions between these factors and time on task (pre vs. post) could not be examined.

Jay et al. [2] examined the influence of sleep disordered breathing (SBD) on night-time sleep and daytime functioning during a 3-day firefighting simulation with 20 volunteer firefighters. Compared to participants without SBD (*n* = 12), those with SBD (*n* = 8) obtained less sleep, their sleep was more disturbed, they felt more fatigued, and they performed more poorly on a reaction time task. These data are consistent with the well-established effects of SBD on night-time sleep and daytime functioning. It is interesting to note that the greatest difference between the groups, in terms of total sleep time, occurred on the first night. This suggests that those with SBD may find it more difficult to adapt to unfamiliar sleep environments than those without SBD.

Sargent et al. [3] conducted a laboratory-based study to examine the impact of time-of-day and circadian phase on feelings of hunger and satiety in 28 healthy, young, male, adults. Participants were divided into two groups with either 6 h in bed per 24 h (moderate sleep restriction, *n* = 14) or 4 h in bed per 24 h (severe sleep restriction, *n* = 14). Both groups lived on a non-24-h ‘forced desynchrony’ schedule, so that the effects of circadian phase could be observed independent of the effects of prior wake. There were no differences in hunger or satiety between the moderate and severe sleep restriction groups. Considering both groups together, hunger and satiety exhibited strong circadian rhythms such that hunger was highest, and satiety was lowest, during body clock evening (i.e., 17:00–21:00 h), whereas hunger was lowest, and satiety was highest, during body clock night-time (i.e., 01:00–05:00 h). These data indicate that the disruption to eating behaviours caused by night work may be mediated by the circadian system rather than the sleep/wake system.

Workplace drowsiness, particularly during night shifts, is a major contributing factor to errors and accidents. Francois et al. [4] validated a system that they have developed to automatically estimate drowsiness based on ocular parameters extracted from images of the eye, i.e., photo-oculography. During 28 h of total sleep deprivation, 24 participants completed a 10-min reaction time test after being awake for ~2 h (09:00–10:00), ~17 h (02:00–03:00), and ~26 h (11:00–12:00). Images of the right eye and polysomnographic signals were recorded during all three reaction time tests. Estimates of drowsiness based on the new photo-oculography algorithms were consistent with standard measures of drowsiness based on reaction time performance and polysomnography. The authors identified a threshold drowsiness score for their photo-oculography system that could detect lapses, i.e., reaction times >500 ms, with sensitivity of 72% and specificity of 80%. If translated to the workplace, this would mean that when drowsiness is present, it is not detected in 28% of cases (false negatives), and when drowsiness is not present, it is incorrectly detected in 20% of cases (false positives). For any fatigue detection technologies such as this to be useful, they must be implemented within a risk management system that allows safety to be maintained when false negatives occur, and ecological validity to be maintained when false positives occur.

Grech [5] reviewed the issues related to controlling fatigue in the maritime industry, which requires continuous 24-h operations over several days, weeks, or months. In shipping, international working time arrangements are regulated through prescriptive rules, such that work cannot exceed 14 h in a 24-h period or 72 h in a 7-day period and rest cannot be less than 10 h in a 24-h period (divided into no more than two periods) or 77 h in a 7-day period. There is good evidence to show that compliance with these work and rest limits is generally poor and that the information that is recorded in logs is not necessarily consistent with actual hours of work and rest. Given the problems associated with this prescriptive approach, including poor compliance and poor record keeping, the author suggests that fatigue may be better controlled in the maritime industry by taking a risk management approach. This is consistent with emerging trends in other long-haul transportation modalities including aviation, trucking and rail.

Ferguson et al. [6] conducted a multiple-day firefighting simulation with 88 volunteer firefighters to identify tests that are sensitive to fatigue outside a controlled laboratory environment. From a range of tests, the Samn-Perelli Fatigue Checklist and the Psychomotor Vigilance Task were the most sensitive subjective and objective measures of fatigue, respectively. Nevertheless, these tests produce a high number of false negatives, i.e., fail to detect fatigue when it is present, so if they are used to control fatigue amongst firefighters, then it should be as tools within a system that has multiple redundant layers of defence against fatigue-related errors and accidents.

Taken together, the papers chosen for this Special Issue provide an excellent insight into current topics in fatigue research that are relevant to public health. First, this Special Issue highlights the fact that working long shifts and/or night shifts can affect not only cognitive functioning, but also physical health. In particular, three papers in this issue examined the potential relationships between shiftwork and different aspects of health, including the cardiovascular system [1], sleep disordered breathing [2], and eating behaviour [3]. Second, this Special Issue highlights the move away from controlling fatigue through prescriptive hours of service rules and toward the application of risk management principles. In particular, three papers indicated that best-practice fatigue risk management systems should contain multiple redundant layers of defence against fatigue-related errors and accidents [4,5,6].
